# Comparison of Cumulative Live Birth Rates between Flexible and Conventional Progestin-Primed Ovarian Stimulation Protocol in Poor Ovarian Response Patients According to POSEIDON Criteria: A Cohort Study

**DOI:** 10.3390/jcm12185775

**Published:** 2023-09-05

**Authors:** Ying Chen, Yifan Chu, Wen Yao, Luyao Wang, Wanjiang Zeng, Jing Yue

**Affiliations:** 1 Reproductive Medical Center, Tongji Hospital, Tongji Medical College, Huazhong University of Science and Technology, Wuhan 430030, China; 2 Department of Obstetrics and Gynecology, Tongji Hospital, Tongji Medical College, Huazhong University of Science and Technology, Wuhan 430030, China

**Keywords:** cumulative live birth rate, progestin primed ovarian stimulation, medroxyprogesterone acetate, ovarian stimulation, in vitro fertilization, POSEIDON criteria

## Abstract

Research Question: To compare the cumulative live birth rate (CLBR) per oocyte retrieval cycle of a conventional progestin-primed ovarian stimulation (cPPOS) regimen with a flexible progestin-primed ovarian stimulation (fPPOS) regimen in poor ovarian response patients, according to POSEIDON criteria. Design: Poor ovarian response women, according to POSEIDON criteria, who underwent the first PPOS protocol for in vitro fertilization/intracytoplasmic sperm injection (IVF/ICSI) between January 2018 and December 2020 were included. The fPPOS group involved 113 participants, and the cPPOS group included 1119 participants. In the cPPOS group, medroxyprogesterone acetate (MPA) (10 mg/d) was administrated on the gonadotropin injection the same day as gonadotropin injections in the cPPOS group, while MPA was started either on the day when the leading follicle with mean diameter > 12mm was present and/or serum E_2_ was >300 pg/mL in the fPPOS protocol group. The primary outcome was CLBR. Results: The fPPOS protocol had higher CLBR per oocyte retrieval cycle compared to the cPPOS group, even without a statistically significant difference (29.6% vs. 24.9%, *p* = 0.365). The fPPOS group had fewer numbers of retrieved oocytes (2.87 ± 2.03 vs. 3.76 ± 2.32, *p* < 0.001) but a higher MII oocyte rate (89.8% vs. 84.7%, *p* = 0.016). In addition, the number of available embryos in the two groups was comparable (1.37 ± 1.24 vs. 1.63 ± 1.38, *p* = 0.095). There were five women in the fPPOS group, and 86 women in the cPPOS group had a premature LH surge (4.2% vs. 6.8%, *p* = 0.261). In the fPPOS group, there was one instance of premature ovulation, while in the cPPOS group, there were six occurrences of premature ovulation (0.8 vs. 0.5%, *p* = 1.000). Conclusion(s): The novel fPPOS protocol appears to achieve higher CLBR even without significant differences and with MPA consumption compared with cPPOS protocol in low-prognosis patients.

## 1. Introduction

Poor ovarian response (POR) is characterized by poor response to standard regimens in assisted reproduction technology (ART), resulting in an insufficient number of retrieved oocytes, a higher cancellation rate, and a lower live birth rate [1]. The Patient-Oriented Strategies Encompassing Individualized Oocyte Number (POSEIDON) criteria proposed in 2016 stratified POR patients into four groups by age, anti-Müllerian hormone (AMH), antral follicle count (AFC), body mass index (BMI), and the number of oocytes in the previous oocyte retrieval cycle [2,3,4]. Women who meet the POSEIDON criteria are presumed to have a lower prognosis after ART treatment than normal responders with the same ovarian reserve [5]. Therefore, how to improve the clinical outcomes of POSEIDON patients remains to be one of the greatest agendas in ART [6].

With the advent of in vitro fertilization and embryo transfer (IVF–ET) treatment, in order to increase the efficacy of the treatment, ovarian stimulation (OS) was introduced to retrieve more oocytes and generate more embryos available for transfer. Nevertheless, multi-follicular growth results in increased production of sex steroids, which can lead to an extemporaneous LH surge and spontaneous ovulation prior to oocyte collection [7]. Thus, pituitary suppression becomes an important research topic of reproductive physiology during controlled ovarian hyperstimulation (COH) for good outcomes in ART [8]. Following gonadotrophin-releasing hormone antagonists (GnRH-ant) protocol and gonadotrophin-releasing hormone agonist (GnRH-a) protocol, experts proposed a progestin-primed ovarian stimulation (PPOS) regimen. The PPOS regimen was proposed by Kuang et al. in 2014 [9], in which progesterone, instead of GnRH analogs, is administered during the follicular phase to prevent the LH surge [9].

The advantages of PPOS are avoiding daily injections and being low-cost. It has been proved that this protocol can obtain competent or better oocytes, embryos, and clinical outcomes compared to other protocols in specific populations [8,10,11,12]. However, fresh cycle transfer cannot be performed in patients who performed the PPOS protocol, which may induce new questions, such as the possible adverse effects of cryopreservation on embryos and offspring. With the further improvement of in vitro culture and freeze–thaw technology, some recent studies have confirmed that the freeze-all approach does not have an effect on cumulative live birth rate (CLBR), but on the contrary, avoids the negative effects of offspring in the state of high estrogen [13,14]. Additionally, the security of the protocol remains in question. Whether high progesterone status, particularly the heavy use of medroxyprogesterone acetate (MPA) in artificial PPOS regimens, negatively affects oocyte quality and offspring safety remains controversial. Based on this, a novel flexible progestin-primed ovarian stimulation (fPPOS) regimen was applied recently [15]. Unlike the conventional PPOS (cPPOS) protocol, oral progestin administration was not started simultaneously with gonadotrophins but was added flexibly according to the follicular size and/or serum E_2_ levels in the fPPOS protocol. Compared to the cPPOS protocol, the dosage of MPA was lower in the fPPOS protocol. Therefore, the fPPOS protocol was more advantageous in terms of security and may be more effective in poor ovarian response patients. Indeed, the safety and effectiveness of the fPPOS protocol have been approved, especially in poor ovarian response patients [16].

As far as we know, no previous study has compared the efficacy and safety of fPPOS protocol and cPPOS protocol in POR patients. Therefore, the purpose of the present study was to investigate whether fPPOS for the treatment of POR patients according to POSEIDON criteria achieved the same or better pregnancy outcomes than those of cPPOS protocol.

## 2. Methods

### 2.1. Study Population

We carried out a retrospective cohort study that included women undergoing in vitro fertilization/intracytoplasmic sperm injection (IVF/ICSI) from January 2018 to December 2020 at the reproductive center of Tongji Hospital, Tongji Medical College of Huazhong University of Science and Technology, Wuhan, China. All data were extracted from the electronic medical record database. This study was approved by the Ethical Committee of Tongji Hospital on 28 March 2021 (TJ-IRB20210528).

**Figure 1:**
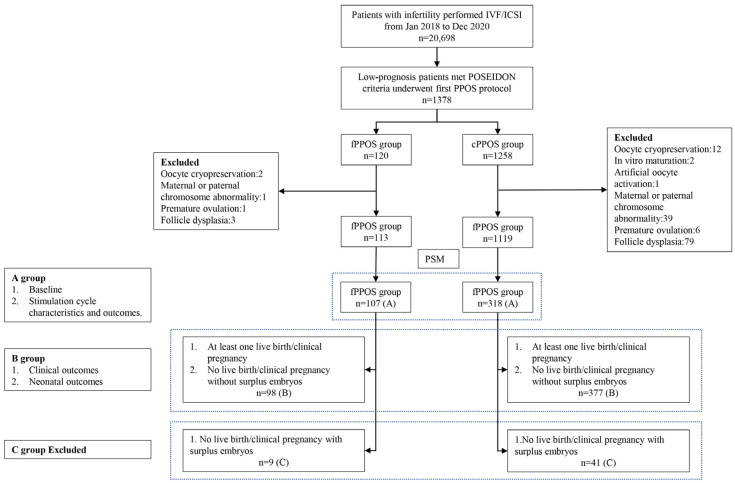
Flow chart of the selection of patients for inclusion in this study. IVF, in vitro fertilization. ICSI, intracytoplasmic sperm injection. POSEIDON criteria, The Patient-Oriented Strategies Encompassing Individualized Oocyte Number criteria. fPPOS, flexible progestin-primed ovarian stimulation protocol. cPPOS, conventional progestin-primed ovarian stimulation protocol. PSM, propensity score matching.

Patients who met the POSEIDON criteria and underwent the first PPOS protocol were enrolled in this study. The POSEIDON criteria were defined as the criteria proposed in 2016 [2]. We further excluded 146 women for (i) oocyte cryopreservation, (ii) in vitro maturation, (iii) oocyte artificial activation, (iv) maternal or paternal chromosome abnormality, and (v) moderate to severe intrauterine adhesions. Finally, 113 women were included in the fPPOS group, and 1119 women were included in the cPPOS group (Figure 1).

### 2.2. Ovarian Stimulation Protocols and Fertilization

As described previously, baseline characteristics were collected on days 2–3 of the menstrual cycle to exclude any follicles > 12 mm and uterine or ovarian pathology [15]. Then, the patients were given gonadotropin daily at a dose of 150–300 IU/day and MPA 10 mg/day daily simultaneously in the conventional group. In the flexible group, MPA at 10 mg/day was started either when the leading follicle with a mean diameter > 12 mm was present and/or serum E_2_ was >300 pg/mL. The Gn doses were adjusted based on the ovarian response assessed by serum hormone levels and the development of follicles by transvaginal ultrasonography every 2–3 days. For both groups, ovulation was triggered with subcutaneous injections of human chorionic gonadotropin (HCG 10,000 IU, Lizhu Pharmaceutical Trading Co. Ltd., Zhuhai, China) when the leading follicle exceeded 17 mm diameter, or the majority growing follicles reached 14 mm or more. Oocytes were retrieved via transvaginal aspiration 34–36 h after ovulation was triggered. Fertilization was carried out in vitro, by either conventional insemination or ICSI, depending on semen parameters. All embryos were vitrified, considering the detrimental endometrial receptivity by MPA.

### 2.3. Transfer of Cryopreserved-Thawed Embryos

Frozen embryo transfer (FET) was performed in the subsequent cycle, as described previously [17,18]. Endometrial preparation protocols included the natural cycle and hormone replacement protocol, which were primarily determined by the patient’s ovulation status [19]. Natural cycles were adopted for patients with regular menstrual cycles. Dydrogesterone was administered orally, and progesterone was administered vaginally on the ovulation day for the luteal phase support. The hormone replacement protocol was adopted for irregular menstrual cycles. Valerate estrogen was orally administered until the endometrial thickness reached a maximum of 7 mm. Luteal phase support was similar to the natural cycle. The maximum number of transferred embryos was two. The progesterone supplementation was continued until 10 weeks of gestation after pregnancy was achieved.

### 2.4. Primary and Secondary Outcomes

The primary outcome is the cumulative live birth rate (CLBR). The secondary outcomes are cumulative pregnancy rate (CPR), the number of oocytes retrieved, MII oocytes, available embryos, the MII oocytes rate, the two pronuclei (2PN) embryos rate, duration of ovarian stimulation, total usage of gonadotropin, the incidence of premature LH surge, cancellation rate, follicular output rate (FORT), follicle-to-oocyte (FOI), and blastocyst rate per patient.

CLBR is defined as the rate of at least one live birth per oocyte retrieval cycle; women with the surplus embryo(s) by the end of an agreed follow-up period (two years) have been excluded [20,21,22]. CPR is defined as the cumulative clinical pregnancy rate per oocyte retrieval cycle or until all embryos are used. The available embryos are defined as cryopreserved embryos. The 2PN embryo rate is calculated as the rate of 2PN embryos to all MII oocytes per woman. The premature LH surge is defined as serum LH > 10 IU/L or rising above twice the basal level before trigger day. The cancellation rate is defined as the rate of cancel cycle due to premature ovulation or follicle dysplasia [9]. Neonatal complications included stillbirth, neonatal jaundice, pneumonia of the newborn, neonatal pulmonary insufficiency, and birth defects [21]. FORT is calculated as pre-ovulatory follicle (>14 mm in diameter) count on dhCG × 100/small antral follicle (3–8 mm in diameter) count at baseline [23]. FOI is defined as the total number of oocytes retrieved/the number of antral follicles available (AFC) [24]. The blastocyst formation rate is calculated as the number of blastocysts/all embryos per patient (B group) per oocyte retrieval. The endpoint is the same as CLBR.

### 2.5. Statistical Analysis

Statistical analysis was performed with SPSS 26.0 software (SPSS, Chicago, IL, USA). The distribution of variables was evaluated visually with histograms. Continuous variables were presented with mean ± (SD) and compared via Student’s *t*-test or Mann–Whitney U Test. Categorical variables were compared with the Chi-square test or Fisher’s exact tests. A two-sided *p*-value < 0.05 was considered statistically significant.

Propensity score is a statistical method used in non-randomized studies to control confounding factors in a non-parsimonious way. Five covariates were selected to estimate the propensity score by unconditional multivariate logistic regression, including age, BMI, AFC, AMH, and infertility type, for their potential to be confounders for the effectiveness of both ovarian stimulation protocols. Women of the fPPOS and cPPOS groups were propensity-matched in a 1:3 ratio without replacement. Since this is a retrospective study, all women stimulated with the fPPOS protocol were included and matched with women stimulated with the cPPOS protocol.

## 3. Results

Between January 2018 and December 2020, there were 1378 women who underwent a PPOS cycle in our reproductive center. Among these, 14 women underwent oocyte cryopreservation, two women underwent in vitro maturation, one woman underwent oocyte artificial activation, and 40 couples had maternal or paternal chromosome abnormalities. Overall, 1232 women who underwent their first PPOS were included (Figure 1). There were nine patients in the fPPOS group and 41 patients in the cPPOS group who did not obtain live birth/clinical pregnancy with surplus embryos, so they were excluded after PSM (8.4% vs. 12.9%, *p =* 0.213).

Among the 1232 women who were included, 1119 underwent a cPPOS protocol, and 113 women underwent a fPPOS protocol. Table 1 shows the demographic, clinical, and IVF treatment characteristics of those patients. Before PSM, significant differences were observed between the two protocols in age, AFC, and infertility type. Subsequent PSM minimized the imbalance of baseline characteristics (Table 1). A total of seven cycles were canceled due to premature ovulation or follicle dysplasia. Of these, one premature ovulation happened in the fPPOS group, and six premature ovulations happened in the cPPOS group (0.8 vs. 0.5%, *p =* 1.000). There were four women in the fPPOS group and 85 women in the cPPOS group who had a premature LH surge without premature ovulation (3.3% vs. 6.8%, *p =* 0.145) (Table 2). 

The fPPOS group had fewer numbers of retrieved oocytes (3.20 ± 2.14 vs. 4.45 ± 2.75, *p <* 0.001), MII oocytes (2.87 ± 2.03 vs. 3.76 ± 2.32, *p <* 0.001), and 2PN embryos, but a higher MII oocytes rate (89.8% vs. 84.7%, *p =* 0.016). The number of available embryos was comparable between the two groups (1.37 ± 1.24 vs. 1.63 ± 1.38, *p =* 0.095). After fertilization in vitro, the 2PN embryo rate, the duration of ovarian stimulation, and total gonadotropin usage (2912.59 ± 1230.01 vs. 2948.25 ± 987.45, *p =* 0.194) were comparable. The total MPA usage of the fPPOS group was significantly lower than the cPPOS group (75.61 ± 29.34 vs. 120.20 ± 23.54, *p <* 0.001) (Table 2). Ovarian sensitivity indexes involve FORT and FOI. FORT was comparable between the two groups (80.31% ± 64.01 vs. 85.26% ± 54.45, *p* = 0.437), whereas FOI was significantly lower in the fPPOS group than the cPPOS group (61.33% ± 32.68 vs. 75.3 ± 30.20, *p* < 0.001). We compared the blastocyst rate of patients in the B group (Figure 1) to reduce bias. The blastocyst rate was comparable in the two groups (19.44% vs. 23.86%, *p* = 0.407). The fPPOS group showed a higher trend of CLBR (29.6% vs. 24.9%, *p =* 0.365) and CPR (37.8% vs. 32.9%, *p =* 0.379) than the cPPOS group, even without a significant difference. In group 1, stratified by POSEIDON criteria, the CLBR of fPPOS was lower than the cPPOS group without a significant difference (15.4% vs. 40.0%, *p =* 0.189), but in the rest of the groups, the CLBR of fPPOS were all higher than cPPOS. In group 4, the CLBR of the fPPOS group was significantly higher than the cPPOS group (34.6% vs. 14.8%, *p =* 0.024) (Table 3).

Of all the pregnancies, 113 women in the fPPOS group and 1119 women in the cPPOS group had 34 and 73 deliveries, respectively. The incidence of twin pregnancy (82.8% vs. 94.2%, *p =* 0.159) was comparable in the two groups. Total neonatal complications (5.9% vs. 9.6%, *p =* 0.788), gestational age (265.48 ± 2.40 vs. 269.10 ± 1.38, *p =* 0.172), and the sex distribution of newborns (32.4% vs. 43.9%, *p =* 0.100) were also similar between the two groups (Table 4).

## 4. Discussion

This is the first clinical study to compare the fPPOS protocol with the cPPOS protocol on CLBR for low-prognosis women who met POSEIDON criteria undergoing IVF/ICSI treatment. Our study showed that the novel fPPOS protocol involves a trend toward higher CLBR with less MPA consumption compared with the cPPOS protocol in low-prognosis patients. In addition, no differences in the incidence of premature ovulation or premature LH surge were detected. The results provided evidence that the fPPOS protocol seems to be a promising choice for aged and low-prognosis patients who met the POSEIDON criteria.

Since the advent of ART, how to improve clinical outcomes of IVF/ICSI in low-prognosis patients has been one of the most important topics. GnRH-ant and GnRH-a [25] are equally recommended for predicted poor responders according to ESHER guidelines [26]. Recently, progestins have been used as an alternative to GnRH analogs for preventing ovulation during ovarian stimulation [27]. High progesterone can inhibit LH peaks when multiple follicles are recruited with exogenous gonadotropin. The fPPOS regimen costs less and can be administered orally, which is more convenient for infertile women. Previously published studies have demonstrated that the PPOS protocol showed comparable oocyte retrieval and pregnancy outcomes compared with other protocols [7,12,28]. Our study focused on a low-response population and confirmed that compared to the cPPOS regimen, the fPPOS regimen can achieve similar clinical outcomes, even with better clinical outcomes in group 4 of the POSEIDON criteria.

The safety of PPOS protocol regarding neonatal outcomes and congenital malformations is still controversial. Several studies have been published recently regarding the safety of the PPOS regimen, indicating that no changes were observed in the newborn congenital malformations of the PPOS regimen and other regimens. The outcomes of our study indicate that the PPOS protocol seems to be an effective choice for reaching competent CLBR. Even though the indicators assessing infants in our study were comparable, the long-term safety of babies is still questioned. A retrospective cohort study included patients with advanced endometriosis induced by GnRH-a, GnRH-ant, and PPOS protocols demonstrated that there were no apparent differences concerning newborn congenital malformations [29]. A meta-analysis involving 9274 live-born infants also showed no detrimental effect detected with PPOS on congenital malformations and low birth weight compared with GnRH-a short protocols [30]. In summary, published studies so far confirmed the safety of the regimen on neonatal outcomes. The patients in the cPPOS group were given gonadotropin and MPA simultaneously, which led to heavy use of progestogens. Researchers have expressed concerns regarding the potential negative impact of high progesterone status on the oocyte retrieved day. Therefore, the fPPOS regimen was proposed in 2019. In the fPPOS regimen, MPA was started depending on the leading follicle size and serum E_2_ level. Compared to the cPPOS regimen, the usage time of MPA was significantly reduced in the fPPOS regimen. Our data showed that the total MPA consumption of fPPOS was significantly less than the cPPOS group (75.61 ± 29.34 vs. 120.20 ± 23.54, *p* < 0.001), which may be more favorable in terms of safety.

An early LH peak or premature ovulation is more prone to occur in the POR patients [31], for decreased activity of gonadotrophin surge-attenuating factor [32,33] may lead to a high cancel rate in IVF/ICSI. A review published recently has proved POR patients receiving the PPOS protocol had fewer canceled cycles compared to those receiving other protocols [33]. However, the studies included in this review were the cPPOS protocol. Additionally, the flexible addition of progestogens may increase the risk of the occurrence of premature LH surge, premature ovulation (0.8 vs. 0.5%, *p* = 1.000), and cancel cycle, but our results showed no difference between the flexible and conventional PPOS protocols. A study has investigated the use of the fPPOS protocol for pituitary suppression, specifically targeting patients with POR [16]. The results showed four instances of premature LH surge among 27 women who underwent the fPPOS protocol, which aligns with our findings. PPOS is recommended to apply before the rise of estrogen levels in the early stage of follicular development. During ovarian stimulation, both estrogen and progesterone levels increase simultaneously. It is advised not to use PPOS for patients with high baseline estrogen levels (E, 70 pg/mL). When the estrogen level is 70 pg/mL, and the follicle diameter is 7–8 mm, it is not recommended to add progesterone due to the risk of early LH surge, which may lead to luteinization [34]. Though the potential mechanism of LH suppression remains to be explored, progesterone can be applied clinically to prohibit estradiol-induced LH surges by blocking the initiation of the GnRH surge induction system [10].

Several studies have suggested that progesterone in PPOS protocol may offer a variety of choices, such as MPA, dydrogesterone (DYG), or dienogest (DNG). Among these studies, one study that compared DNG and DYG reported a reduced number of oocytes in the DNG group [35]. Three studies comparing DYG, MPA, and progesterone reported that MPA displayed effective blocking effects in premature LH surges [36,37,38]. MPA is the most commonly used treatment among them. The effective dosage has also raised concerns. Two studies compared different dosages of MPA (4 vs. 10 mg/day), and one of them reported similar oocytes retrieved outcomes, while another one reported better clinical outcomes in the 10 mg/d group [8,39]. In our study, MPA (10 mg/day) was chosen, and the results showed that MPA administration in PPOS protocol produces comparable clinical outcomes [35,36,37,38,40,41,42]. However, further randomized controlled trials are [35,36,37,38] warranted to confirm this conclusion.

Interestingly, the number of retrieved oocytes and MII oocytes was lower significantly in the fPPOS group, which was consistent with ovarian sensitivity indexes (FORT and FOI) results. Sara Cesarano et al. reported that the FORT index is of high accuracy in predicting the number of MII oocytes. The FOI index demonstrated a statistical correlation with both the number of MII oocytes and the success of embryonic culture [43]. However, both indexes are not statistically predictive for CLBR. After embryonic culture, the available embryos were comparable between the two groups. Based on the findings of this study, the fPPOS group achieved a trend toward higher CPR and CLBR (29.6% vs. 24.9%, *p* = 0.365) than the cPPOS group overall. A study published by Zhang et al. reported a similar CLBR (25.2%) in low-prognosis women who received the PPOS protocol with other protocols [28]. Then, the patients were stratified into four groups by POSEIDON criteria. It is reported that a minimum of 6–9 and 10–15 mature oocytes are required to obtain one euploid blastocyst for transfer in POSEIDON groups 3 and 4. CLBR of the fPPOS group was lower in group 1 (age < 35 years with normal ovarian reserve) but higher in group 2 (aged ≥ 35 years with normal ovarian reserve), group 3 (age < 35 years with poor ovarian reserve), and group 4 (aged ≥ 35 years with poor ovarian reserve), proving fPPOS regimen may be more suitable for low-prognosis women [44]. A multicenter retrospective cohort study showed that POSEIDON patients have a lower prognosis than normal responders [4,45]. Therefore, stimulation regimens should be chosen more carefully. The CLBR of the fPPOS group was lower in group 1 (age < 35 years with normal ovarian reserve) but higher in group 2 (aged ≥ 35 years with normal ovarian reserve), group 3 (age < 35 years with poor ovarian reserve), and group 4 (aged ≥ 35 years with poor ovarian reserve), proving that the fPPOS regimen may be more suitable for low-prognosis women [44]. Nevertheless, the accuracy of outcomes was limited by the sample size. Larger sample size studies could further illuminate these questions. These observations speak against any harmful effect of fPPOS protocol on pregnancy outcomes.

There are still several limitations in the present study. First, it was a retrospective cohort study conducted in a single center, and the limited sample size may introduce a selection bias. Then, the health condition of neonatal was not followed up, and the negative effect on neonatal outcomes and congenital malformations in children born with fPPOS protocol has not been revealed. Therefore, more prospective multicenter studies with a larger number of patients need to be conducted in the future.

In conclusion, it appears that the fPPOS protocol is better than the cPPOS protocol, considering the higher probability of pregnancy in low-prognosis women. The premature LH surge rate and premature ovulation rate support that the fPPOS protocol does not hamper the effect of pituitary suppression in low-prognosis women undergoing ovarian stimulation. Further studies are warranted to confirm these findings, especially with neonatal outcomes and congenital malformations in POR populations.

## Figures and Tables

**Table 1:** Demographic, clinical, and IVF treatment characteristics of women in the fPPOS group and the cPPOS group.

Outcome Measure	Before Propensity Score Matching			After Propensity Score Matching		
	fPPOS	cPPOS	*p* Value	fPPOS	cPPOS	*p* Value
NO. of patients	113	1119		107	318	
Age, years	34.10 ± 4.87	36.61 ± 5.75	<0.001	34.40 ± 4.77	34.29 ± 5.00	0.834
BMI, kg/m^2^	22.22 ± 2.93	22.28 ± 2.85	0.887	22.23 ± 2.92	22.19 ± 2.96	0.423
AFC	5.01 ± 2.21	4.12 ± 1.90	<0.001	4.83 ± 2.13	4.75 ± 1.87	0.702
AMH, μg/mL	1.17 ± 0.85	1.10 ± 0.83	0.271	1.17 ± 0.87	1.17 ± 0.88	0.983
Basal FSH, mIU/mL	10.58 ± 4.71	10.80 ± 5.71	0.867	10.53 ± 4.76	10.75 ± 5.51	0.717
Cause of infertility (%)						
Male	1/113 (0.90)	12/1119 (1.10)	0.853	0/107 (0)	6/318 (1.90)	0.330
Female	83/113 (73.5)	841/1119 (75.2)	0.690	79/107 (73.8)	237/318 (76.0)	0.659
Mix	28/113 (24.8)	266/1119 (22.9)	0.811	27/107 (25.3)	75/318 (24.0)	0.804
Others	1/113 (0.90)	0/1119 (0)	0.092	1/107 (0.90)	0/11119 (0)	0.252
Infertility type (%)						
Primary infertility	72/113 (63.7)	587/1119 (52.5)	0.022	68/107 (63.6)	209/318 (65.7)	0.683
Secondary infertility	41/113 (36.3)	532/1119 (47.5)		39/107 (36.4)	109/318 (34.3)	
Infertility time	3.65 ± 3.57	3.58 ± 3.26	0.732	3.63 ± 3.65	3.66 ± 2.96	0.920
Fertilization methods (%)						
IVF	82/113 (72.6)	743/1119 (66.4)	0.184	64/107 (59.8)	185/318 (58.2)	0.766
ICSI	31/113 (27.4)	376/1119 (33.6)		43/107 (40.2)	133/318 (41.8)	

Values are presented as mean ± SD or proportion (%); AMH, anti-Müllerian hormone; AFC, antral follicle count; BMI, body mass index.

**Table 2:** Stimulation cycle characteristics and outcomes.

Outcome Measure	fPPOS	cPPOS	*p* Value
No. of oocytes	3.20 ± 2.14	4.45 ± 2.75	<0.001
No. of MII oocytes	2.87 ± 2.03	3.76 ± 2.32	<0.001
No. of 2PN embryos	1.93 ± 1.62	2.43 ± 2.01	0.022
No. of available embryos	1.37 ± 1.24	1.63 ± 1.38	0.095
MIIoocyte rate (%)	307/342 (89.8)	1197/1414 (84.7)	0.016
2PN embryo rate (%)	207/307 (67.4)	767/1197 (64.1)	0.273
Duration of stimulation, days	9.64 ± 2.10	9.29 ± 2.08	0.134
Total usage of gonadotropin, IU	2912.59 ± 1230.01	2948.25 ± 987.45	0.194
Total usage of MPA, IU	75.61 ± 29.34	120.20 ± 23.54	<0.001
FORT (%)	80.31 ± 64.01	85.26 ± 54.45	0.437
FOI (%)	61.33 ± 32.68	75.34 ± 30.20	<0.001
Premature LH surge rate (%)	5/120 (4.2)	86/1258 (6.8)	0.261
Premature ovulation rate (%)	1/120 (0.8)	6/1258 (0.5)	1.000
Cancellation rate (%)	4/120 (3.3)	85/1258 (6.8)	0.145

MII, metaphase II. 2PN, two pronuclear fertilized. LH, luteinizing hormone.

**Table 3:** Comparison of clinical outcomes from fPPOS and cPPOS protocols.

Outcome Measure	fPPOS	cPPOS	*p* Value
CPR (%)	37/98 (37.8)	91/277 (32.9)	0.379
CLBR (%)	29/98 (29.6)	69/277 (24.9)	0.365
1	2/13 (15.4)	18/45 (40.0)	0.189
2	6/20 (30.0)	7/34 (20.6)	0.652
3	12/39 (30.8)	31/110 (28.2)	0.759
4	9/26 (34.6)	13/88 (14.8)	0.024

CPR, cumulative pregnancy rate per oocyte retrieval cycle. CLBR, cumulative live birth rate per oocyte retrieval cycle.

**Table 4:** Comparison of neonatal outcomes from fPPOS and cPPOS protocols.

Outcome Measure	fPPOS	cPPOS	*p* Value
Infant number	34	73	
P1 (%)	24/29 (82.8)	65/69 (94.2)	0.159
P2 (%)	5/29 (17.2)	4/69 (5.8)	
Gender (%)			
Female	11/34 (32.4)	36/73 (43.9)	0.100
Male	23/34 (67.6)	37/73 (56.1)	
Gestational age	265.48 ± 2.398	269.10 ± 1.377	0.172
Very preterm (%)	0	0	0.291
Preterm (%)	7/29 (24.1)	9/69 (13.0)	
Term (%)	22/29 (75.9)	60/69 (87.0)	
Neonatal complications in total, %	2/34 (5.9)	7/73 (9.6)	0.788

P1, pregnant with one newborn infant. P2, pregnant with two newborn infants.

## Data Availability

The data presented in this study are available on request from the corresponding author. The data are not publicly available due to privacy.
